# A High-Temperature Superconducting Bandpass Dual-Mode Filter with Tunable Relative Bandwidth and Center Frequency

**DOI:** 10.3390/s23031079

**Published:** 2023-01-17

**Authors:** Zhaojiang Shang, Liejun Shen, Jianyong Huang, Huibin Zhang

**Affiliations:** College of Sciences, Shanghai Institute of Technology, Shanghai 201418, China

**Keywords:** high-temperature superconducting (HTS), bandpass filter (BPF), varactor, dual-mode resonator (DMR), tunable

## Abstract

This study proposes a high-temperature superconducting (HTS) bandpass filter with a continuously tunable bandwidth and center frequency. The proposed filter combines several gallium arsenide varactors and a dual-mode resonator (DMR). The even and odd modes of the DMR can be tuned simultaneously using a single bias voltage. The capacitive value of varactors in the circuit is tuned continuously under continuous voltage and frequency tunability. External couplings and the interstage can be realized using an interdigital coupling structure; a fixed capacitor is added to the feeder to improve its coupling strength. A low-insertion loss within the band is obtained using HTS technology. Additionally, the proposed filter is etched on a 0.5 mm-thick MgO substrate and combined with YBCO thin films for demonstration. For the as-fabricated device, the tuning frequency range of 1.22~1.34 GHz was 9.4%; the 3-dB fractional bandwidth was 12.95~17.39%, and the insertion loss was 2.28~3.59 dB. The simulation and experimental measurement results were highly consistent.

## 1. Introduction

Filters are crucial in modern wireless communication systems (WCSs). Over the past decades, various filters have been proposed and thoroughly investigated [1,2,3]. Recently, extensive studies on filters have been conducted, resulting in the rapid development of modern WCSs, which generally run at multiple standards and frequencies. Particularly, filters with electronic tunability and reconfigurability are crucial in modern WCSs because they can reduce cost and system complexity [4,5,6,7]. Filters with microwave tenability are crucial for WCSs both presently and in the future. Semiconductor varactors have been widely used in developing tunable filters owing to their cost effectiveness, high tuning speed, and miniaturized size [8,9,10,11]. High-temperature superconducting (HTS) filters that are characterized by reduced insertion loss, steep band edge, and increased out-of-band rejection are promising for these applications [12]. Recently, HTS varactor-tuned filters, characterized by dual-mode resonators (DMRs) [13] and an open-loop [14], have been proposed. Additionally, these filters exhibit tunable bandwidths owing to frequency tuning. However, few studies on high-order dual-mode tunable filters have been reported owing to complicated bias circuits and high-insertion loss.

In this study, a four-pole HTS-tunable filter consists of two DMRs, which can be applied to WCSs. The resonator can be tuned simultaneously using a single bias voltage (SBV). Meanwhile, external couplings and interstage can be realized using an interdigital coupling structure, and a fixed capacitor is added to the feeder to improve the coupling strength. In addition, the HTS-tunable filter is simulated by Sonnet. The tunable filter was fabricated on a 0.5 mm-thick MgO substrate with YBCO films, and the relative dielectric constant (RDC) of the substrate was 9.8. The circuit dimension was 21.6 mm × 13 mm, and it was mounted on a metal carrier and fabricated on a metal shield box. By changing the SBV, the center frequency variable range of the HTS-tunable filter is 1.22~1.34 GHz. The 3-dB fractional bandwidth increased from 12.95% to 17.39%. The results of simulations and experimental measurements are highly consistent.

## 2. Analysis of DMRs

**Figure 1:**
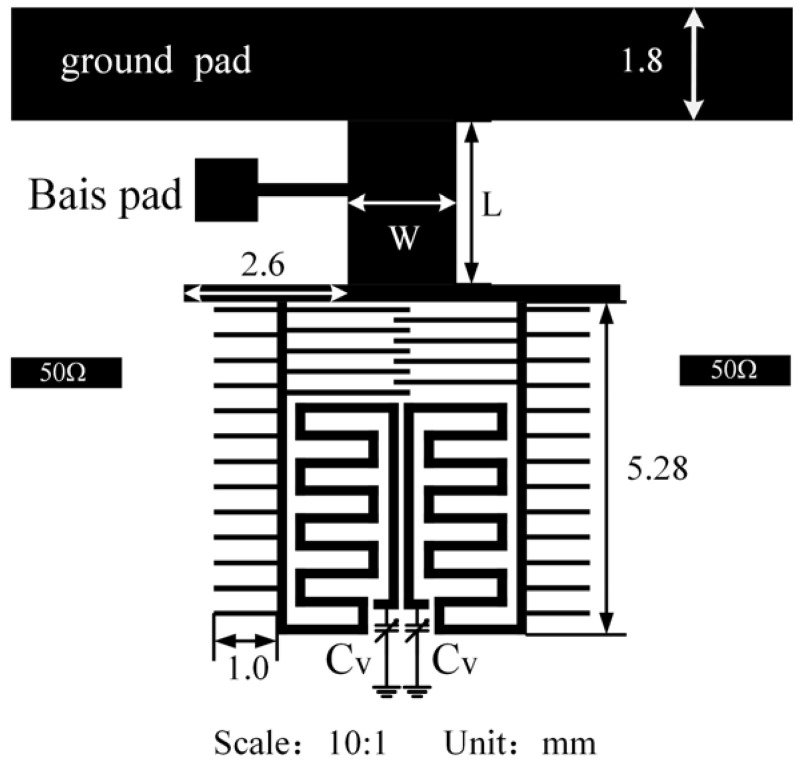
The structure of the proposed tunable resonator.

The proposed resonator is shown in Figure 1. The resonator comprises four parts: the middle part denotes the length of the uniform microstrip line. A short-circuited stub (SCS) is located above the microstrip line, which is connected to a bias circuit. The microstrip line is loaded below the two finger-containing open-circuited stubs (OCS), and the end of the OCS is loaded with a semiconductor varactor *C_v_*. Additionally, the grounding of the short transect is realized using superconducting grounding pads (GPs).

**Figure 2:**
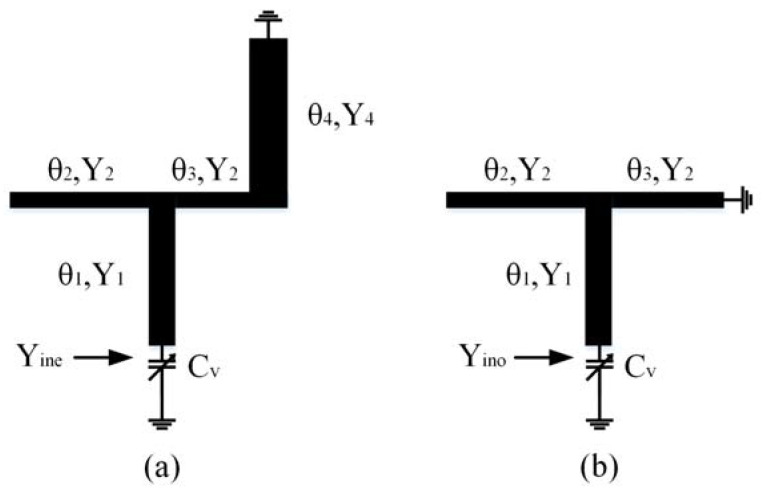
A simplified equivalent circuit model of the dual-mode resonators (DMR) proposed in this study. (**a**) Even mode; (**b**) odd mode.

The resonance characteristics of this resonator can be investigated using the even–odd mode method owing to symmetrical characteristics. The influence of the finger line on the open end is ignored for simplicity of analysis. The odd- and even-mode equivalent circuits are shown in Figure 2a and Figure 2b, respectively.

For the even mode of the resonator shown in Figure 2a, the input admittance *Y_ine_* can be deduced as

(1) $$Y_{ine}=j\left(Y_{e}+\omega C_{v}\right)\;$$

(2) $$Y_{e}=Y_{1}\frac{1-\tan\theta_{2\;}tan\theta_{3}-k_{2}p_{1}\;tan\theta_{4}-p_{2}\;tan\theta_{1}}{tan\theta_{1}\;tan\theta_{2}\;tan\theta_{3}+k_{2}p_{1}\;tan\theta_{1}\;tan\theta_{4}-tan\theta_{1}-p_{2}}$$

where *k_*1*_ = Y*_1_*/Y*_2_, *k*_2_ = *Y*_2_/*Y*_4_, *k*_3_ = *Y*_1_/*Y*_4_, *p_*1*_* = *tanθ*_2_ + *tanθ*_3_, *p*_2_ = *k*_1_ *tanθ*_3_ + *k*_3_ *tanθ*_4_. The odd-mode input admittance *Y_ino_* shown in Figure 2b can be deduced as

(3) $$Y_{ino}=j\left(Y_{o}+\omega C_{v}\right)\;$$

(4) $$Y_{o}=Y_{1}\frac{1-\tan\theta_{2\;}tan\theta_{3}-k_{1}\;tan\theta_{1}\;tan\theta_{3}}{tan\theta_{1}\;tan\theta_{2}\;tan\theta_{3}-tan\theta_{1}-k_{1}\;tan\theta_{3}}$$

where *k*_1_ = *Y*_1_/*Y*_2_. When *Y_ino_* = 0 and *Y_ine_* = 0 are applied, the odd- and even-mode resonance conditions can be obtained.

As shown in Figure 2, *C_v_* influences the even- and odd-mode frequencies. Therefore, the odd- and even-mode frequencies can be adjusted using a variable capacitor only. This simplifies the adjustment of the filter frequency. Additionally, the SCS (*Y*_4_, *θ*_4_) influences the even-mode frequency only, as shown in Figure 2, but not the odd-mode frequency. This can be used to conveniently adjust the odd and even modes to the desired frequency.

**Figure 3:**
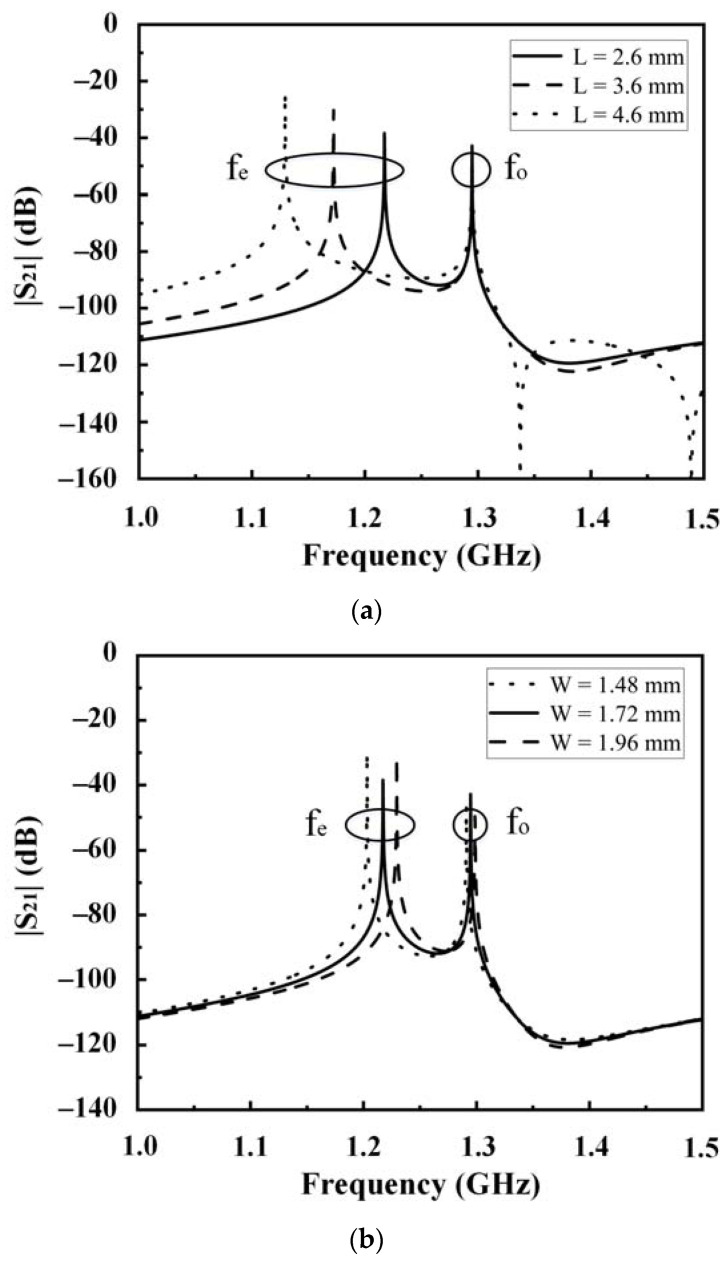
Modal resonant characteristics with varied microstrip line lengths. (**a**) L; (**b**) W.

The frequency response of the resonator is analyzed under weak coupling conditions by using Sonnet. The modal resonance characteristics at different microstrip line lengths of the resonator (L and W) are shown in Figure 3. Herein, f_o_ and f_e_ represent odd and even mode frequencies, respectively. As shown in Figure 3, the position of f_o_ remains unchanged with a variation in W and L, as long as the other variables remain constant. However, f_e_ decreases as L increases, and f_e_ increases as W increases. In other words, the odd- and even-mode frequencies can be controlled separately, which can be easily tuned to the desired frequency band. Therefore, the bandwidth of the filter can be easily controlled. This is consistent with findings from previous studies.

## 3. Design of a Four-Pole HTS-Tunable Filter

**Figure 4:**
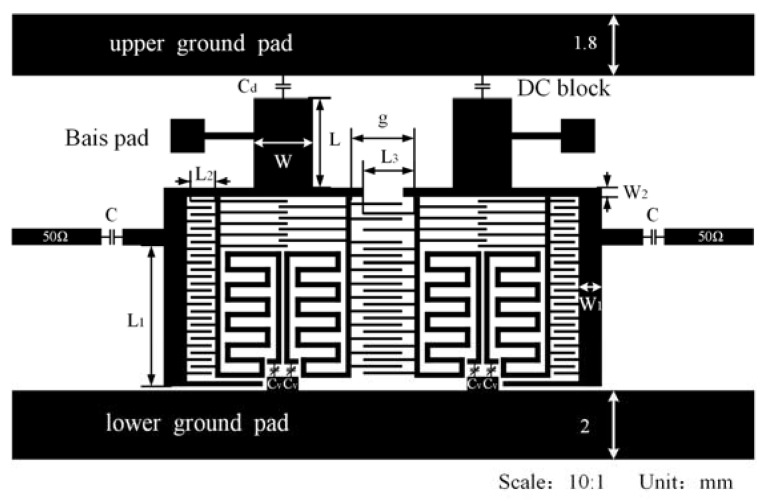
The proposed high-temperature superconducting (HTS) tunable bandpass filter with four poles.

The proposed HTS-tunable filter with four poles is shown in Figure 4. The symmetrical microstrip filter comprised two DMRs, bias circuits, input/output feedlines, direct current (DC) blocking capacitors, two GPs, fixed capacitors, and varactors. The proposed filter is on an MgO substrate with a thickness of 0.50 mm and a RDC of 9.8. Herein, the shunt resonator is implemented using a foldable half-wavelength transmission line that is loaded with *C_v_* at each end of the OCS. Interdigital coupling is employed for inter-resonator coupling. The finger length is adjusted to achieve the designated coupling. The input/output feedlines, with fixed capacitors C, are coupled to the DMR to achieve the designated external coupling. Additionally, four transmission zeros are introduced into the upper stopband and one transmission zero into the lower stopband to improve out-of-band suppression. Meanwhile, the two GPs (upper and lower) provide radio frequency (RF) and DC grounding, wherein RF signals are shorted using the DC blocking capacitors (C_d_), whereas DC signals are blocked using the C_d_. The DC bias pads are connected to the SCSs of the DMRs using a high-impedance line to avoid leakage of RF signals.

The grounding technique is essential for the design of an HTS-tunable filter. The resonant frequency is influenced by the HTS GP, which is influenced by its width. The upper HTS GP shown in Figure 4 is designed with a dimension of 21.6 × 1.8 mm^2^, whereas the lower HTS GP has a size of 21.6 × 2 mm^2^ with negligible impact on the RF performance. The soldering bias pad, with dimensions of 1 × 1 mm^2^, is connected to the resonator. This connection is achieved using a high-impedance line with a width of 0.2 mm.

*C_v_* decreases as the applied bias voltage (BV) increases. Thus, the center frequency of the filter increases as BV increases. Once the initial resonator structure is determined, the coupling control mechanism is investigated to satisfy the requirements of the design parameters (e.g., internal coupling coefficients *k*, external quality factor *Q_e_*) for the particular specifications in the passband.

The coupling configuration proposed is shown in Figure 4. According to [11], *k* at the resonant frequencies may be calculated as

(5) $$k=\frac{f_{2}^{2}-f_{1}^{2}}{f_{2}^{2}+f_{1}^{2}}\;$$

where *f_1_* and *f*_2_ refer to lower and upper coupling resonant frequencies, respectively.

**Figure 5:**
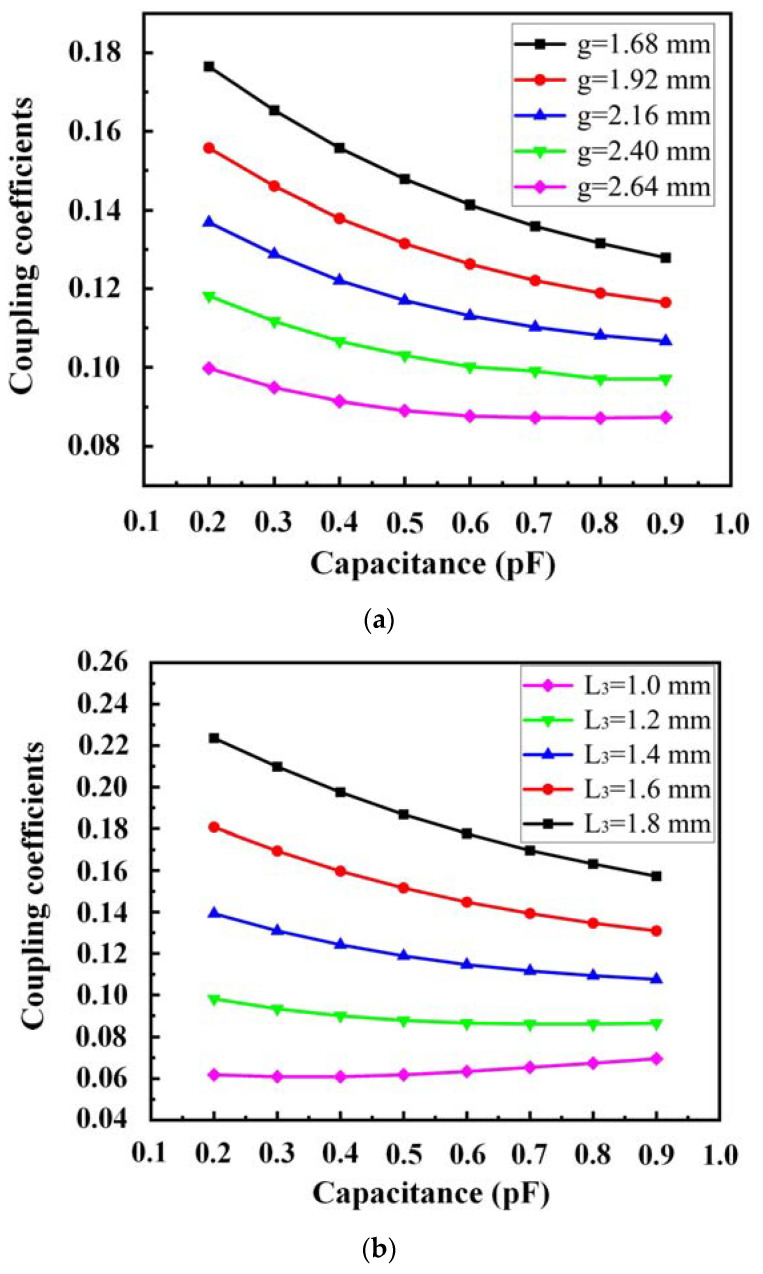
Coupling coefficients as a function of the coupling distance, varying with different *C_v_*. (**a**) g; (**b**) L_3_.

The initial distances, in this case, are g = 1.92 mm and L_3_ = 1.48 mm. *k*, a function of g and L_3_, and *C_v_* are obtained by tuning the varactor's value with the others maintained constant. The results are shown in Figure 5.

The coupling coefficient when *C_v_* = 0.2~0.9 pF under other variables is shown in Figure 5. According to Figure 5a, when *C_v_* remains constant, the *k* of the resonators decreases with increasing g. As shown in Figure 5b, when *C_v_* remains constant, the *k* of the resonators increases with increasing L_3_.

Compared with g, L_3_ significantly influences *k*. Herein, L_3_ is determined, and *k* is then fine-tuned by g. Therefore, the value of *k* can satisfy the desired value by selecting appropriate parameters.

**Figure 6:**
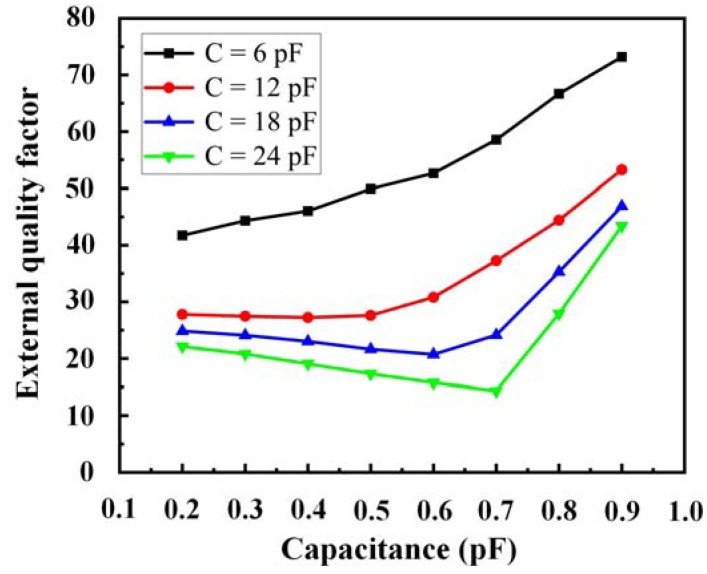
Variations in the simulated *Q_e_* with *C_v_* for different values of C.

A pair of fixed capacitors C is connected to the input and output matching networks to adjust the external coupling. The external quality factors can be calculated as follows [11]:

(6) $$Q_{e}=\frac{f_{0}}{\Delta f_{3dB}}\;$$

where *f*_0_ refers to the resonance frequency, and *Δf*_3*dB*_ refers to the 3 dB bandwidth. As shown in Figure 6, C is adjusted such that *C_v_* has a suitable *Q_e_* ranging from 0.2 to 0.9 pF. The external coupling of the filter can be easily adjusted by changing the capacitance value of C. By changing the capacitance value of C, the value of the external coupling can be appropriately changed, so that the filter can work normally under different center frequencies.

## 4. Simulation and Measurement Results

**Figure 7:**
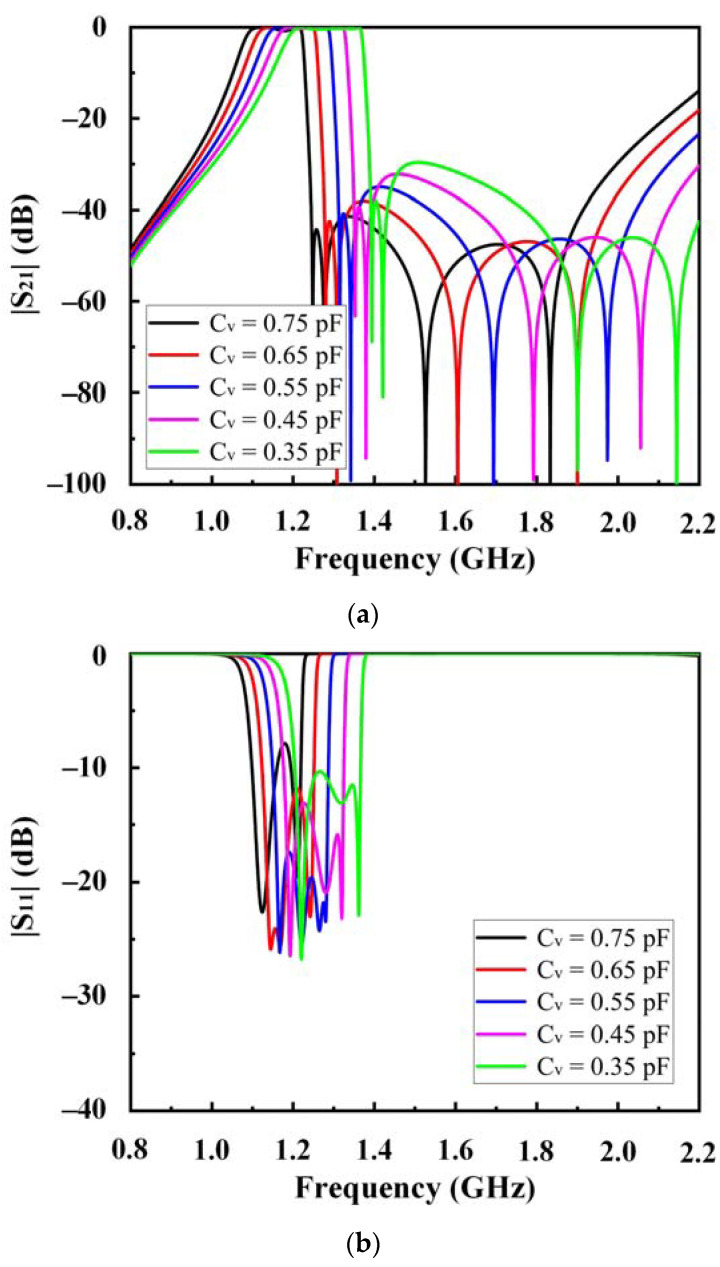
Simulated. (**a**) S_21_; (**b**) S_11_ of the four-pole HTS-tunable filter.

The four-pole HTS-tunable filter, whose relative bandwidth increased with increasing tunable frequencies, was simulated using the Sonnet. The circuit dimensions shown in Figure 4 are summarized in Table 1. Figure 7 shows the simulation S-parameter curve of the HTS-tunable filter. The simulation results show that when the varactor varies in the range of 0.9~0.2 pF, the center frequency of the passband varies in the range of 1.11~1.33 GHz (adjustable range of 18%), and the relative bandwidth increases with the increase in the adjustable frequency (increased from 12.07 to 16.09%). The return loss of the passband exceeded −9 dB in most cases.

**Figure 8:**
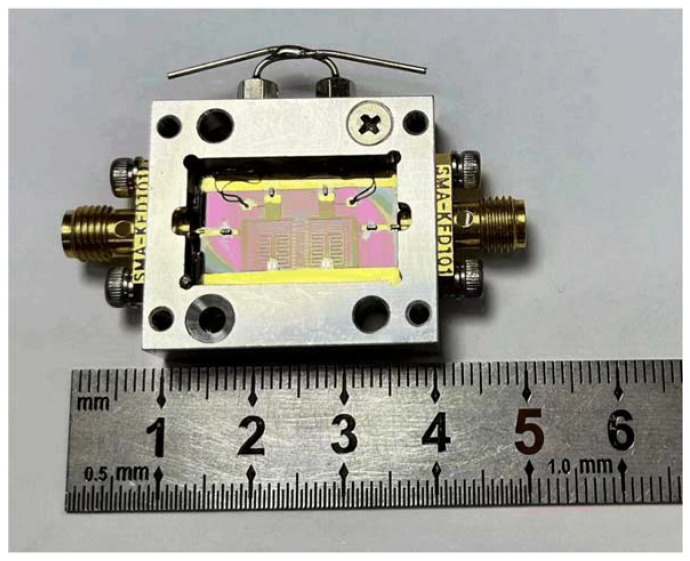
Image of the as-fabricated HTS bandpass filter with connectors.

Particularly, the tunable filter was fabricated on a 0.5 mm-thick MgO substrate with YBCO films, and the RDC of the substrate was 9.8. The circuit dimension was 21.6 mm × 13 mm, and it was mounted on a metal carrier and fabricated on a metal shield box. Varactors (M/A-COM MA46H120), 18 pF fixed capacitors, and 100 nF DC blocking capacitors, characterized by miniaturization and high Q-factor, were mounted to the circuit using silver epoxy glue. An image of the as-prepared four-pole filter is shown in Figure 8. Application of the DC bias of each *C_v_* to the circuit was achieved using the power supply via feed-through capacitors. Additionally, a fixed capacitor C was placed between the feeders to tune *Q_e_*. The varactor capacitances were determined using a pre-fabricated HTS-tunable filter that ranged from 0.9 to 0.2 pF, whereas the reverse BV ranged from 1~13 V.

**Figure 9:**
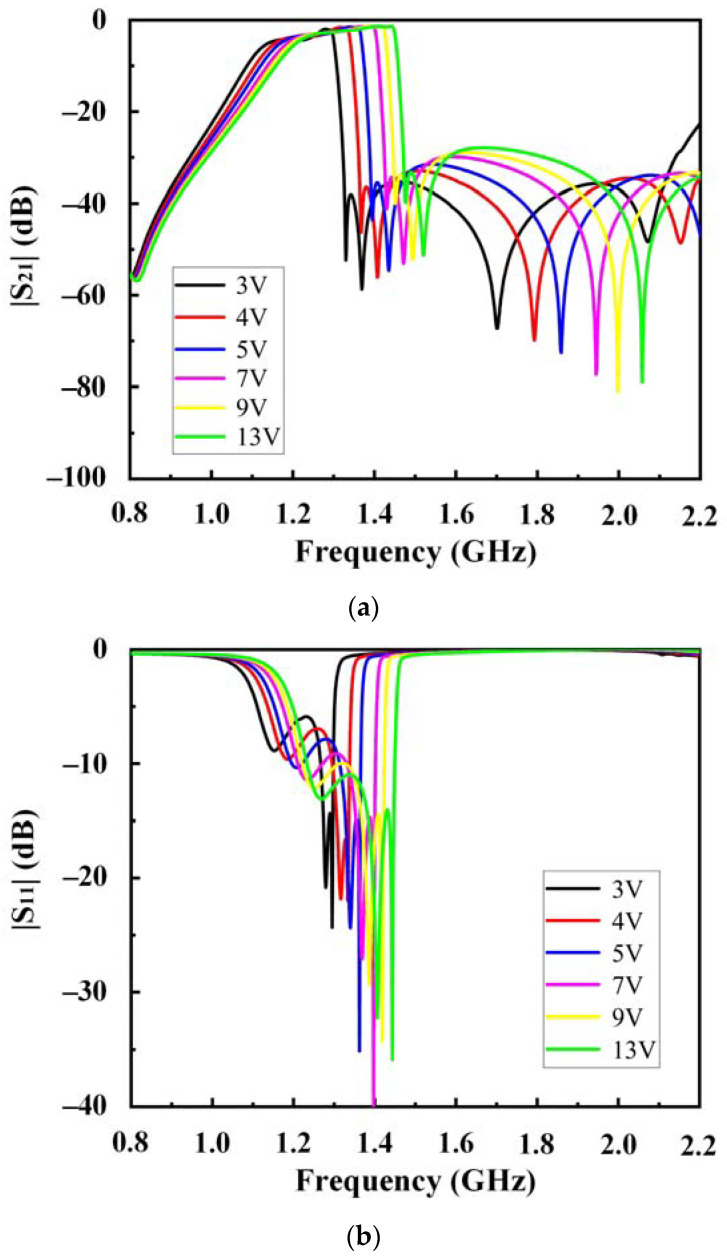
Measured (**a**) S_21_; (**b**) S_11_ of the four-pole HTS-tunable filter.

The filter was cooled to 77 K. The results of the experimental measurements are shown in Figure 9. The experimental measurement results in cases of different BVs are listed in Table 2. The filter frequency can be tuned in the range of 1.22~1.34 GHz. The passband maximum insertion loss was approximately 3.59 dB at 3 V. However, the insertion loss at the lower side was higher than that at the upper side. This can be attributed to the DC blocking capacitors, which influenced the even mode (but not the odd mode) at lower frequencies, as shown in Figure 2 and Figure 3. The measured bandwidth is a little wider than the simulated one. This may be caused by DC block capacitors, fixed capacitors, and varactors. The decrease of in-band return loss leads to enlarged bandwidth. Despite the bias state, the return loss of the passband exceeded −8 dB in most cases. As presented, the 3 dB fractional bandwidth increased with increasing bias. As BV increased from 3 to 13 V, the 3 dB fractional bandwidth increased from 12.95 to 17.39%. The proposed filter was compared with several varactor-tunable microstrip filters in terms of performance, as shown in Table 3. The proposed filter exhibited a relatively small insertion loss and larger fractional bandwidth than others.

## 5. Conclusions

A four-pole compacted HTS-tunable filter was implemented using a DMR. Four transmission zeros were generated in the upper stopband, and one transmission zero was generated in the lower stopband using a feedline structure. The filter frequency was tuned by applying an SBV to four varactors. The rationality of the circuit design was validated through simulations and experiments, and the HTS-tunable filter, whose bandwidth increased with tunable frequencies, was fabricated. The proposed filter had a high filtering performance and excellent suitability for multi-service communication applications.

## Figures and Tables

**Table 1:** Circuit dimensions of the as-fabricated filter (unit: mm).

Parameters	W	W_1_	W_2_	L	L_1_	L_2_	L_3_	g
**Dimensions (mm)**	1.72	0.68	0.28	2.6	4.08	0.72	1.48	1.92

**Table 2:** Experimental measurement results of the as-fabricated filter.

No.	Bias Voltage/V	Frequency /GHz	3-dB Bandwidth /MHz	Insertion Loss/dB
1	3	1.22	158	3.59
2	4	1.25	175	3.50
3	5	1.27	189	3.10
4	7	1.30	208	2.64
5	9	1.32	219	2.42
6	13	1.34	233	2.28

**Table 3:** Performances of the proposed and other filters.

Ref.	Filter Order	Tuning Range/MHz	C /pF	Insertion Loss/dB	3-dB Fractional Bandwidth
[15] [16]	4	830–1010	2.7–3.3	3–4	3–3.6%
	4	1550–2100	0.45–2.5	4–6.5	2.2–8% (1 dB)
[17]	4, HTS	430–720	0.5–20	0.8–3.8	2.8–3.2%
[5]	2	1700–2700	0.3–2.4	3.8–4.9	2.9–6.5% (1 dB)
[18]	4	3750–4000	0.72–4.15	<6 dB	3.5–7%
[11]	4, HTS	73.2–118	1–21	0.6–2.2	3.2–4.3%
this work	4, HTS	1220–1340	0.2–0.9	2.28–3.59	12.95–17.39%

## Data Availability

The data presented in this study are available on request from the corresponding author.
